# MRI‐based surrogates of brain clearance in narcolepsy type 1

**DOI:** 10.1111/jsr.14479

**Published:** 2025-02-18

**Authors:** Eva M. van Heese, Jari K. Gool, Gert Jan Lammers, Ysbrand D. van der Werf, Matthias J. P. van Osch, Rolf Fronczek, Lydiane Hirschler

**Affiliations:** ^1^ Department of Anatomy and Neurosciences Amsterdam UMC, Vrije Universiteit Amsterdam Amsterdam the Netherlands; ^2^ Amsterdam Neuroscience, Neurodegeneration Amsterdam the Netherlands; ^3^ Sleep‐Wake Centre, Stichting Epilepsie Instellingen Nederland (SEIN), Heemstede the Netherlands; ^4^ C. J. Gorter MRI Center, Department of Radiology Leiden University Medical Centre Leiden the Netherlands; ^5^ Amsterdam Neuroscience, Compulsivity, Impulsivity and Attention Amsterdam the Netherlands; ^6^ Department of Neurology Leiden University Medical Centre Leiden the Netherlands

**Keywords:** brain clearance, DTI‐ALPS, glymphatics, hypersomnolence, MRI, narcolepsy type 1

## Abstract

Brain clearance involves the drainage of waste molecules from the brain, a process that is suggested to be amplified during sleep. Recently proposed MRI‐based methods attempt to approximate human brain clearance with surrogate measures. The current study aimed to explore whether two brain clearance surrogates are altered in narcolepsy. We processed diffusion‐weighted and functional resting‐state images to extract two surrogates: Diffusion Tensor Imaging Along the Perivascular Space (DTI‐ALPS index), and dBOLD‐CSF coupling. Both measures were analysed in 12 drug‐free, awake people with narcolepsy type 1 and 11 age‐ and sex‐matched controls, as well as in relation to clinical features. We also assessed the correlation between the DTI‐ALPS index and dBOLD‐CSF coupling. The DTI‐ALPS index and dBOLD‐CSF coupling amplitude did not show significant differences between narcolepsy and controls, nor significant relations with the severity of excessive daytime sleepiness. We found a significant correlation between dBOLD‐CSF coupling and sleep efficiency, as well as a significant correlation between the DTI‐ALPS index and dBOLD‐CSF coupling. The hypothesis of altered brain clearance in narcolepsy type 1 is not supported by evidence from the current study. The two surrogates correlated with each other, suggesting that both offer different perspectives from the same underlying physiology. Yet, the suitability of the surrogates as brain clearance markers remains debatable. Whereas DTI is not exclusively sensitive to perivascular fluid, dBOLD‐CSF coupling is reflecting large‐scale CSF motion. Future work should explore other surrogate markers, preferably during sleep, to better understand the possible role of altered brain clearance in narcolepsy type 1 symptomatology.

## 1 INTRODUCTION

### 1.1 Narcolepsy

Narcolepsy type 1 is a primary neurological sleep disorder characterised by excessive daytime sleepiness (EDS), disturbed nocturnal sleep, and cataplexy (i.e. involuntary loss of muscle tone induced by strong emotions). As people suffering from narcolepsy experience strongly reduced daytime functioning and quality of life, it poses a significant burden for patients, partners, and society (Jennum et al., 2012). The underlying pathophysiology of narcolepsy type 1 is deficiency of hypocretin‐1, a neuropeptide involved in sleep–wake regulation that is normally mainly produced in the lateral hypothalamus (Nishino et al., 2000; Thannickal et al., 2000). From the hypothalamus, hypocretin‐producing cells have widespread projections to the whole brain, with dense connections to the brainstem nuclei and basal forebrain (Ebrahim et al., 2002). In narcolepsy, wakefulness is highly disrupted by sleep‐related processes such as lapses in attention, drowsiness, and unintended naps, offering a unique opportunity to study the effects of disrupted wakefulness on brain function.

### 1.2 Brain clearance

Research on brain clearance has recently gained popularity in the context of healthy sleep and sleep disorders. One of the important elements of the brain clearance system involves cerebrospinal fluid (CSF) movement along channels surrounding the blood vessels: the perivascular spaces. These channels are proposed to be the main pathways for CSF‐mediated clearance. Brain clearance is suggested to be amplified during sleep based on observations of faster amyloid‐β clearance and interstitial fluid (ISF) compartment enlargements in sleeping mice, and a larger CSF compartment volume in sleeping humans (Demiral et al., 2019; Xie et al., 2013). Moreover, sleep deprivation in humans has been shown to slow down brain clearance (studied using tracer enrichment after intrathecal injections; Eide et al., 2021). Currently, different models exist to describe the anatomy and direction of fluid movement underlying brain clearance (Bakker et al., 2019; Carare et al., 2008; Iliff et al., 2012; Zhao et al., 2022).

### 1.3 Altered brain clearance in narcolepsy?

There are several lines of evidence suggesting the presence of brain clearance alterations in narcolepsy. First, the hypothesis of altered clearance is supported by narcolepsy being characterised by a highly disrupted sleep–wake cycle (i.e. high number of awakenings, altered sleep stage transitions, more time spent in stage 1 non‐rapid eye‐movement [NREM] sleep, and fragmented NREM sleep; Pizza et al., 2015; Roth et al., 2013). Brain clearance is suggested to be amplified during NREM sleep, when slow oscillations are linked to changes in vascular dilation. These slow waves are differentially present, with irregularly timed waves in stage 2 and abundant, characteristic slow waves in Stage 3. It has been hypothesised that isolated slow waves (N2) drive CSF more effectively than continuous slow waves (N3) due to the slower vascular response (Lewis, 2021). Fultz et al. (2019) found significantly greater CSF signal power during N2 sleep compared with N1 and wake, but did not investigate N3 due to a lack of continuous N3 sleep being present in the data. Overall, the specific contributions of different sleep stages to brain clearance remain unclear, partly due to the scarcity of evidence and the challenge of mapping animal NREM stages directly to human sleep stages. We hypothesise that the disrupted sleep–wake cycle in narcolepsy (including disturbed nocturnal sleep with frequent awakenings, rapid REM sleep, daytime napping) may impair brain clearance, based on studies showing that sleep plays a critical role in brain clearance (Fultz et al., 2019; Xie et al., 2013) and that severe disturbances, such as sleep deprivation, can impair this process (Eide et al., 2021).

Second, abnormal levels of CSF biomarkers support the idea of altered clearance in narcolepsy. Spinal CSF measurements are often performed in people with narcolepsy, to confirm hypocretin‐1 deficiency. Several studies have used this opportunity to investigate other CSF biomarkers, mostly neurodegenerative markers such as amyloid‐β, phosphorylated and total tau, and neurofilament light. A summary of this work is presented in Table 1 (Baiardi et al., 2020; Heier et al., 2014; Jennum et al., 2017; Kallweit et al., 2012; Liguori, Placidi, et al., 2016; Liguori, Placidi, Albanese, et al., 2014; Lozano‐Tovar et al., 2024; Shimada et al., 2020; Strittmatter et al., 1996; Wang et al., 2021). Whereas the CSF marker deviations in narcolepsy are bidirectional, most evidence points towards lower levels in spinal CSF of amyloid‐β, phosphorylated and total tau compared with healthy controls (Jennum et al., 2017; Liguori, Placidi, et al., 2016; Liguori, Placidi, Albanese, et al., 2014). It is also hypothesised that the lowest levels of amyloid‐β are present at disease onset if it is triggered by inflammation, and that long‐term treatment with psychostimulants might induce neuronal metabolic changes resulting in higher levels of neurodegenerative markers in the CSF (Liguori, Placidi, Izzi, et al., 2014). Whereas, lower levels of waste molecules in spinal CSF could suggest waste accumulation in the brain as a result of impaired clearance, it remains unclear whether lower marker levels in people with narcolepsy are related to altered brain clearance.

**TABLE 1:** CSF marker studies in narcolepsy.

Study	Population (sample size)	CSF markers	Main results
Baiardi et al. (2020)	NT1 (36) vs controls (30)	Aβ40, Aβ42, Nfl, *p*‐tau, t‐tau	No differences found
Heier et al. (2014)	NT1_[normal hypocretin]_ (18) vs controls (17) NT1_[low hypocretin]_ (18) vs controls (17) NT1_[low hypocretin]_ (18) vs NT1_[normal hypocretin]_ (18)	Aβ42, NSE, *p*‐tau, t‐tau	**Higher** Aβ42, NSE, *p*‐tau, t‐tau in NT1_[normal hypocretin]_ **Higher** Aβ42, NSE, *p*‐tau, t‐tau in NT1_[low hypocretin]_ **Lower** Aβ42 in NT1_[low hypocretin]_
Jennum et al. (2017)	NT1 (10) vs controls (12) NT2 (5) vs controls (12) IH (6) vs controls (12)	Aβ, α‐syn, CHI3L1, Nfl, *p*‐tau, t‐tau	**Lower** Aβ, *p*‐tau, t‐tau in NT1 **Lower** Aβ in NT2 No differences found for IH
Kallweit et al. (2012)	NT1 (2)^b^	Aβ42	Case 1: **Lower** Aβ42 (152 mg/L, whereas >500 mg/L is normal) Case 2: **Lower** Aβ42 (0.000275 mg/L, whereas >500 mg/L is normal)
Liguori et al. (2014)^a^	NT1‐NT2 (16) vs controls (16)	Aβ42, *p*‐tau, t‐tau	**Lower** Aβ42 in NT1‐NT2
Liguori et al. (2016)	NT1‐NT2_[short disease duration]_ ^c^ (13) vs controls (17) NT1‐NT2 _[long disease duration]_ (13) vs controls (17) NT1‐NT2 _[short disease duration]_ (13) vs NT1‐NT2 _[long disease duration]_ (13)	Aβ42, *p*‐tau, t‐tau	**Lower** Aβ42 in NT1‐NT2 _[short disease duration]_ **Lower** Aβ42 and **higher** *p*‐tau in NT1‐NT2 _[long disease duration]_ **Lower** Aβ42 and **lower** *p*‐tau in NT1‐NT2 _[short disease duration]_
Lozano‐Tovar et al. (2024)	NT1 (17) vs controls (91)^d^ NT2 (23) vs controls (91)	Aβ42, *p*‐tau, t‐tau	**Lower** Aβ42, *p*‐tau, t‐tau in NT1 **Lower** Aβ42, *p*‐tau, t‐tau in NT2
Shimada et al. (2020)	NT1 (14) vs controls (17)	268 metabolites	**Higher** histidine in NT1 **Lower** histamine in NT1
Strittmatter et al. (1996)	NT1 (6) vs controls (12)	5‐HIAA, HVA, somatostatin, substance P, VMA	**Lower** 5‐HIAA, somatostatin, substance P, VMA in NT1 **Higher** HVA in NT1
Wang et al. (2021)	NT1 (106) vs controls (51) NT2 (16) vs controls (51) IH (27) vs controls (51)	L‐PGDS^e^	**Higher** L‐PGDS in NT1 **Higher** L‐PGDS in NT2 **Higher** L‐PGDS in IH

Abbreviations: Aβ40, amyloid‐β 1–40; Aβ42, amyloid‐β 1–42; CHI3L1, chitinase 3‐like protein‐1; 5‐HIAA, 5‐hydroxyindoleacetic; HVA, homovanillic acid; IH, idiopathic hypersomnia; L‐PGDS, lipocalin‐type prostaglandin D synthase; Nfl, neurofilament light; NSE, neuron‐specific enolase; NT1, narcolepsy type 1; NT2, narcolepsy type 2; *p*‐tau, phosphorylated tau; α‐syn, α‐synuclein; t‐tau, total tau; VMA, vanillyl mandelic acid.

^a^ Earlier published as abstract under Albanese et al. (2013).

^b^ Case report, values were compared with normal range instead of control group.

^c^ Cut‐off for short‐long disease was 5 years after onset of the first symptom.

^d^ This study also found higher levels of Aβ42 and lower levels of *p*‐tau and t‐tau in narcolepsy compared with Alzheimer's disease.

^e^ An endogenous sleep‐inducing factor.

Third, the findings from MRI diffusivity studies in people with narcolepsy, which identified widespread diffusivity alterations, further hint towards possible fluid motion changes in narcolepsy. Specifically, a lower measure of diffusion directionality (fractional anisotropy), a higher measure of axon myelination (radial diffusivity), and unaffected measures of axon organisation and mean diffusion strength (axial diffusivity, mean diffusivity) were identified in people with narcolepsy compared with controls (Gool et al., 2019; Juvodden et al., 2018; Park et al., 2016; Tezer et al., 2018). The diffusivity alterations may suggest white matter alterations of lower myelination, lower axonal density, or greater axonal diameter, but this was not fully confirmed in post‐mortem histology experiments (Gool, De Brouwer, et al., 2024). It remains unclear to what extent myelin and axonal integrity are affected in narcolepsy type 1, or whether other factors contribute to the diffusivity differences (Gool, Dang‐Vu, & van der Werf, 2024). We consider the possibility that these diffusivity alterations are related to brain clearance alterations in narcolepsy, for instance through larger perivascular spaces and their contribution to the diffusivity measures.

### 1.4 MRI‐based surrogates of brain clearance

Neuroimaging studies have applied contrast‐enhanced MRI to monitor tracer clearance and to study the human brain clearance system in vivo. Other MRI methods, such as adaptations of arterial spin labelling and diffusion‐weighted imaging, have been pursued to image brain clearance with a non‐invasive approach (as summarised in van der Thiel et al., 2023).

Taoka and colleagues (Taoka et al., 2017) proposed a post‐processing technique to be applied to diffusion tensor images: diffusion tensor image analysis along the perivascular space (DTI‐ALPS, Figure S1). The DTI‐ALPS index aims to reflect diffusion along the perivascular space as a measure of brain clearance and has been applied widely in a diverse range of clinical populations (>140 publications in the past 5 years). The index is calculated based on diffusivity in a small area of the brain with a unique, perpendicular position of two fibre tracts and blood vessels, next to the lateral ventricles. Neurodegenerative disorders in which protein accumulations play a central role, such as Alzheimer's and Parkinson's disease, showed lower DTI‐ALPS indices compared with healthy controls (Cai et al., 2023; Chang et al., 2023; Chen et al., 2021; Ma et al., 2021; Taoka et al., 2017). Of note, the index and what it claims to reflect has been disputed as DTI‐ALPS is not CSF‐ nor ISF‐selective and is only measured at a single location in the brain. Changes in DTI‐ALPS index could therefore also originate from changes in tissue pulsatility, changes in white matter structure or the presence of more fluid in the brain (i.e. enlarged PVS, free water due to demyelination, white matter hyperintensities; Taoka et al., 2024; Ringstad, 2024).

Another non‐invasive method allows investigation of the coupling between neurovascular‐driven fluctuations of the blood volume in the cortical grey matter with CSF oscillations at the level of the fourth ventricle (Fultz et al., 2019). An anti‐correlation is observed between cortical grey matter activity (BOLD signal) and CSF waves in awake participants, which is significantly enhanced during sleep. The coupling between the two signals is explained by the following mechanism: activity fluctuations in the cortex give rise to cerebral blood volume changes, which in turn push CSF out, or allow for CSF inflow, due to the restricted space inside the skull (Monroe‐Kellie doctrine). Altogether this dBOLD‐CSF coupling reflects how cerebral blood volume changes can be a driving force for CSF mobility. The dBOLD‐CSF coupling method has been applied in healthy people during sleep (Fultz et al., 2019), and Alzheimer's disease (Han, Chen et al., 2021) and Parkinson's disease during wake (Wang et al., 2023; Han, Brown, et al., 2021).

### 1.5 MRI‐based surrogates of brain clearance in narcolepsy

In narcolepsy, the DTI‐ALPS index has been investigated twice. Gumeler et al. (2023) found no significant group differences, while Hu et al. (2024) identified a lower DTI‐ALPS index, bilaterally, in narcolepsy type 1 compared with controls. Significant correlations of the index with clinical features (polysomnography, sleepiness scales) were described in both studies. The dBOLD‐CSF coupling approach has not been performed in narcolepsy, but showed stronger coupling during sleep compared with wake in healthy volunteers (Fultz et al., 2019).

Altogether, the question remains whether brain clearance is altered in narcolepsy type 1, and whether this can be reliably studied using MRI‐based surrogates of brain clearance. The current study aimed to investigate changes in the DTI‐ALPS index and dBOLD‐CSF coupling in narcolepsy type 1 during wake. We expected to see impaired, weaker brain clearance (lower DTI‐ALPS index, weaker dBOLD‐CSF coupling) in people with narcolepsy type 1 compared with controls, and in relation to markers of disease severity.

## 2 METHODS

### 2.1 Participants

The dataset consisted of 12 people with drug‐free narcolepsy type 1 and 11 healthy, age‐ and sex‐matched controls. Recruitment of people with narcolepsy was carried out through our tertiary sleep clinic (Sleep–Wake Centre SEIN). Participants were diagnosed with narcolepsy type 1 according to the international classification of sleep disorders, third edition (American Academy of Sleep Medicine, 2014). The polysomnography and multiple sleep latency test (MSLT) were performed for the diagnostic process and were not linked to the MRI scan. In this cohort, we observed a diagnostic delay, which is commonly seen in narcolepsy type 1 (median: 50 months, IQR: 34–215 months). The variability in the time between EDS onset and MRI allows for a good representation of patients at both sub‐acute and chronic stages of the disorder (median: 83 months, IQR: 70–311 months). As part of regular care, CSF hypocretin‐1 levels were available for nine out of 12 people with narcolepsy, and all were shown to be deficient according to the narcolepsy type 1 cut‐off of 110 pg/mL (Mignot et al., 2002). Three people with narcolepsy did not present with clear cataplexy and received a lumbar puncture to determine hypocretin‐1 levels, which appeared deficient for two out of three. The third person showed lower levels than typical (138 pg/mL) and was still included based on the presentation of a typical clinical phenotype of narcolepsy. All participants with narcolepsy were HLA DQB1*0602‐positive. Participants were excluded if they (1) used psychotropic drugs; (2) were diagnosed with other serious medical conditions; (3) showed macroscopic brain abnormalities; (4) had contraindications to receive an MRI scan. All individuals had to (1) be between 18 and 65 years old; (2) have normal or corrected‐to‐normal vision; and (3) be right‐handed according to the Edinburgh Handedness Inventory (Oldfield, 1971). Medication use (*n* = 7 drug‐naïve; *n* = 4 methylphenidate; *n* = 1 modafinil) was interrupted for at least 2 weeks and no caffeine‐containing substances were consumed in the 24 h prior to the MRI session. The Dutch Adult Reading Test (proxy for intelligence; Schmand et al., 1991) and Epworth Sleepiness Scale (ESS; Johns, 1991) were administered on the day of the MRI scan. All participants signed informed consent and the study protocol was approved by the medical ethical committee of Leiden University Medical Center. Experiments were conducted in accordance with the declaration of Helsinki.

### 2.2 Image acquisition

All participants were scanned using a 3 T Philips Achieva MRI scanner (Philips Healthcare, Best, the Netherlands) with a 32‐channel head coil and sponge cushions to minimise head motion. Diffusion images were collected with a single‐shot, spin‐echo EPI sequence (2 × 2 × 2 mm^3^ voxel size) with TR = 6700 ms; TE = 72 ms; FOV = 224 × 224 × 120 mm; flip angle = 90°; acquisition time = 5.9 min. This protocol was executed twice with reversed k‐space readout (phase‐encoding in anterior–posterior, AP, and posterior–anterior, PA, direction) along 46 non‐collinear directions with b = 1000 s/mm^2^ and b = 0 s/mm^2^. We acquired 3D T1‐weighted images (1 × 1 × 1 mm^3^) with TR = 8.2 ms; TE = 3.8 ms; TI = 670.4 ms; FOV = 240 × 240 × 220 mm^3^; flip angle = 8°; acquisition time = 6.2 min. Resting‐state (rs) functional images (2.5 × 2.5 × 2.5 mm^3^ voxel size) were acquired with TR = 2250 ms; TE = 29.94 ms; FOV = 200 × 200 × 104.25 mm; flip angle = 80°; 315 dynamics, acquisition time = 11.8 min. Concurrent high‐density EEG was acquired during the rs‐fMRI scan to objectively verify wakefulness. EEG acquisition using carbon‐wire loops and pre‐processing was performed according to methods described in earlier work (van der Meer et al., 2016a, 2016b). To reduce associated artefacts, the helium pump was temporarily switched off. Cleaned EEG recordings were sleep scored according to the AASM criteria using 30‐second epochs.

### 2.3 Diffusion tensor image processing and DTI‐ALPS index

Diffusion image corrections were performed using the FMRIB Software Library's Diffusion Toolbox (FSL, v5.0; Andersson et al., 2003; Andersson & Sotiropoulos, 2016) as described in a previous study within the same dataset (Gool et al., 2019). Tensor fitting was run with the *‐‐save tensor* optional argument to extract the full diffusion tensor for the purpose of this study (DTIFIT, FSL v6.0.6.4). The DTI‐ALPS method was performed as described by Taoka et al. (2017) (Figure S1). For each participant, four 3 × 3 voxel ROIs were drawn on the diffusion tensor ellipsoid maps by one rater, based on the following instructions: (1) use the sagittal and transverse image to find the most superior part of (or just above) the lateral ventricle, this is where most of the deep medullary veins are located in a perpendicular position to the fibres; (2) try different levels of the sagittal plane to find a location with clear separation of the projection (blue) and association (green) fibres; (3) draw the ROIs in areas with clear head‐foot (projection) and anterior–posterior (association) fibre directions based on the ellipsoid shape. No supporting images were available to better identify the deep medullary veins, such as susceptibility‐weighted images. FSLutils commands were applied to extract the diffusivities in three directions of the diffusion tensor, xx, yy, and zz directions for the four ROIs (xx, yy, zz refer to the subject‐specific coordinate system of the diffusion tensor). The DTI‐ALPS index was calculated unilaterally for each participant, and for both images with opposing AP and PA phase‐encoding directions, according to the following formula (1). We tested for differences between the left and right hemisphere, and between the AP and PA phase‐encoding directions, to average in case of non‐significant differences. To extend evidence in the current debate on what the DTI‐ALPS index reflects, we aimed to provide quality control measures on the index, such as left–right comparisons and its relation to the dBOLD‐CSF coupling amplitude. Step‐by‐step description of our approach and code to extract diffusivities and calculate the DTI‐ALPS index are available from github.com/evavanheese/DTI-ALPS.

(1) $$\mathrm{DTI}\;\text{ALPS index}=\text{mean}\left({\mathrm{Dxx}}_{\text{proj}},{\mathrm{Dxx}}_{\text{assoc}}\right)/\text{mean}\left({\mathrm{Dyy}}_{\text{proj}},{\mathrm{Dzz}}_{\text{assoc}}\right)$$

### 2.4 Functional image preprocessing and dBOLD‐CSF coupling

#### 2.4.1 BOLD signal

The resting‐state functional images were slice‐, time‐, and motion‐corrected using the HALFpipe pipeline for harmonised fMRI analysis (Waller et al., 2022; github.com/HALFpipe/HALFpipe). HALFpipe also transformed the output images to MNI standard space, according to the most current and detailed template available (MNI152NLin2009cAsym; Horn, 2016). Cortical grey matter segmentation was performed on the T1‐weighted images using FreeSurfer recon‐all (FS5.3; Fischl et al., 2002). The transformation matrix from the T1‐weighted image to MNI standard space was applied to the cortical grey matter mask (using ANTs registration tools) to extract the average time series from the mask in the same standard space (using FSLutils).

#### 2.4.2 CSF signal

As there is no blood in the CSF ROIs, the signal retrieved from them is not a BOLD signal. In fact, the CSF signal in this study originates from the inflow effect, stating that fresh inflow of CSF, that has not been exposed to radiofrequency (RF) pulses flowing into a slice, has a higher signal intensity than the surrounding tissue in that slice which has been exposed to many RF pulses. With each upward slice, the fluid signal intensity decays until it reaches a steady state, similar to the surrounding tissue (Fultz et al., 2019). Therefore, edge slices are the ideal locations for the most precise measurement of CSF oscillations. Raw functional images were used to extract the signal, as motion correction can alter voxel slice position and performing such corrections correctly on edge slices is complex (Fultz et al., 2019). The first slice was rated in terms of motion and the anatomical position classified into three locations with guidance of the T1‐weighted image (the CSF was either at the level of the fourth ventricle, at the level of the lower cerebellum, or within the central canal). Free‐form CSF ROIs composed of connecting voxels were drawn manually on the bottom slice of the functional image and ranged from 3 to 11 voxels in size. The influence of the anatomical location of the CSF ROI on the dBOLD‐CSF coupling amplitude was investigated in an explorative analysis. A step‐by‐step description of our approach and code to create a cortical grey matter mask based on FreeSurfer output, perform registrations, and extract the BOLD and CSF signal from the ROIs are available from github.com/evavanheese/BOLD-CSF.

#### 2.4.3 Signal processing and cross‐correlation

The BOLD and CSF signals were normalised, detrended, and low‐pass filtered (<0.1 Hz). We then calculated the negative derivative of the BOLD signal (dBOLD), reflecting the change in BOLD signal (i.e. slope of the signal), and set negative values to zero to better reflect inflow and not outflow of CSF (Fultz et al., 2019). To cross‐correlate both signals, we tested different time lags from −20 to 20 seconds and retrieved the lag and amplitude of the peak (positive or negative) closest to the lag of zero seconds. Time courses, as well as cross‐correlation plots, were visually inspected for each subject to confirm a coupling of the CSF with dBOLD signal. Besides the coupling, fluctuations in CSF and dBOLD time series were further investigated by taking the area under the curve (AUC) of the CSF and dBOLD signal time courses. This experiment was done to investigate possible alterations in both signals separately, in addition to the coupling between them. Matlab scripts for signal processing and cross‐correlation are available upon request.

### 2.5 Covariates and statistical analysis

A preregistration of our DTI‐ALPS and dBOLD‐CSF analysis plan and hypotheses was submitted to the Open Science Framework prior to conducting any of the analyses (https://osf.io/mpnat). The primary outcome measures for statistical analysis in this study were: left, right, and bilateral average of the DTI‐ALPS index, and dBOLD‐CSF coupling amplitude and lag. We investigated these outcome measures in relation to EDS (evaluated by the ESS) for several reasons; (1) because it is a hallmark symptom of narcolepsy, (2) because it reflects the intrusion of sleep‐related processes into the wake state, possibly relating to brain clearance, (3) and because it has been linked to brain clearance before (Carvalho et al., 2018). Group differences and correlations with the ESS (*n* = 23), PSG total sleep time, sleep efficiency, and percentage of slow wave sleep (*n* = 12) were investigated using regression models in R (R Core Team, 2014). Given the substantial delay between PSG and MRI data collection (range: 6–106 months) and the small sample size (*n* = 12), these correlations are considered exploratory. Cohen's *D* and Pearson's *r* effect sizes were respectively calculated for each statistical test. All data points were investigated in relation to a range of three times the interquartile range (IQR), and classified as outliers if they exceeded this range. No outliers were identified for the primary outcome measures.

## 3 RESULTS

### 3.1 Final sample

The data analysis was performed on 12 people with narcolepsy type 1 and 11 healthy controls. An overview of the demographic and clinical characteristics from the full sample can be found in Table 2.

**TABLE 2:** Participant characteristics.

	NT1 (*n* = 12)	HC (*n* = 11)	*p* value
Sex (male: female)	8:4	7:4	0.879
Age (years)			
Mean ± SD	33.25 ± 10.50	31.82 ± 13.39	0.777
IQ score (mean ± SD)	110.58 ± 10.73	111.30 ± 8.25	0.865
EDS			
Age of onset (in years; mean ± SD)	19.42 ± 9.15	‐	
Duration (in years; median, IQR)	10.00 (6.00–25.25)	‐	
Cataplexy presence	9/12	‐	
Cataplexy presence and/or hypocretin deficient^a^	12/12	‐	
HLA DQB1*0602 presence	12/12	‐	
ESS score^b^ (mean ± SD)	10.08 ± 3.00	2.64 ± 1.96	**0.001**
Current use of medication	5/12^c^		
MSLT			
Sleep latency (in min; mean ± SD)	4.62 ± 3.64	‐	
SOREM periods (mean ± SD)	2.58 ± 1.57	‐	
Polysomnography			
TST (in min; mean ± SD)	426.50 ± 25.33	‐	
Sleep efficiency (% of TST; mean ± SD)	91.89 ± 5.49	‐	
Stage 1 sleep (% of TST; mean ± SD)	12.75 ± 7.19	‐	
Stage 2 sleep (% of TST; mean ± SD)	43.91 ± 10.38	‐	
Stage 3–4 sleep (% of TST; mean ± SD)	17.40 ± 8.65	‐	
Stage REM sleep (% of TST; mean ± SD)	23.86 ± 6.00	‐	
PLMI (mean ± SD)	4.84 ± 7.50	‐	

*Note*: Significant *p*‐values (<0.05) are displayed in bold.

Abbreviations: EDS, excessive daytime sleepiness; ESS, Epworth Sleepiness Scale; HLA, human leukocyte antigen; MSLT, multi sleep latency test, SOREM, sleep‐onset rapid eye movement; TST, total sleep time; PLMI, periodic leg movement index.

^a^ The hypocretin‐1 level of one patient (138 pg/mL) was slightly higher than the diagnostic threshold (< 110 pg/mL). However, the level can still be classified as deficient and the patient showed a typical clinical phenotype of NT1. Therefore, the patient was included in the study.

^b^ The ESS was obtained on the day of the MRI scan.

^c^ All five drug‐using patients refrained from use of their stimulants (4× methylphenidate, 1× modafinil) for at least 14 days prior to the MRI scan.

### 3.2 DTI‐alps

**FIGURE 1:**
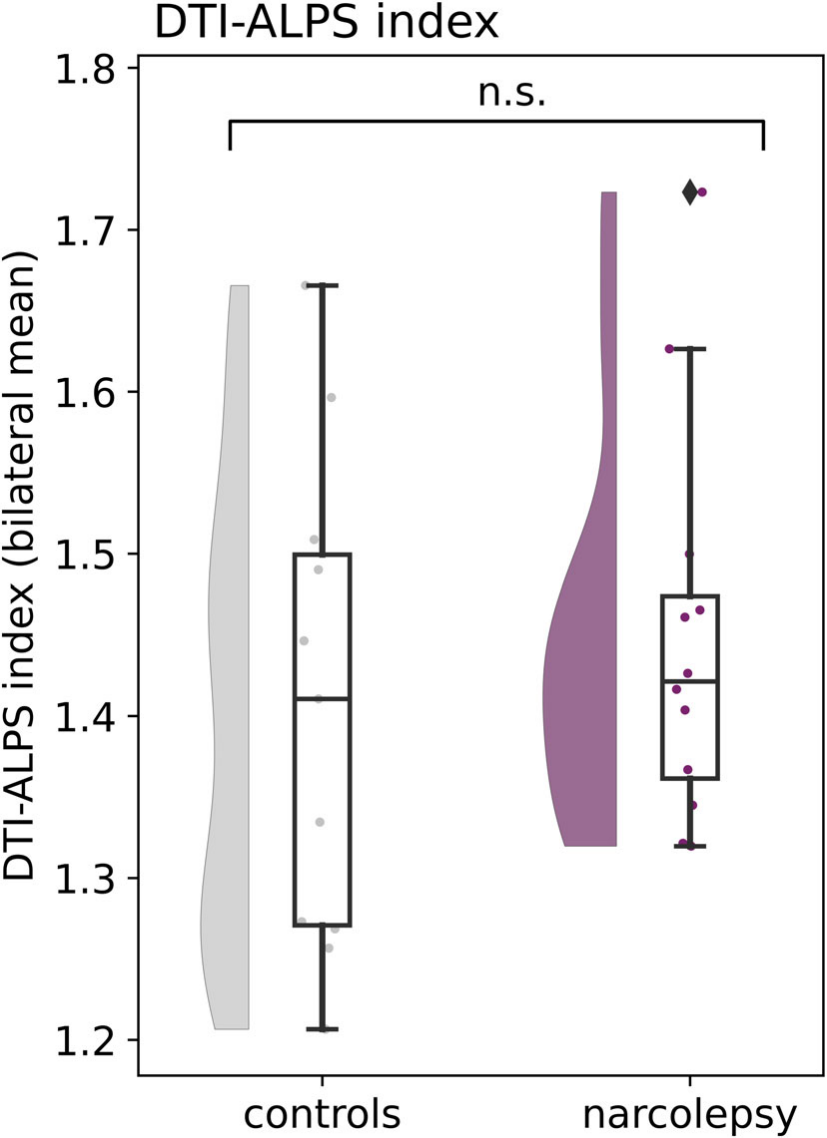
DTI‐ALPS index group differences for the left and right hemisphere. No significant differences were found between the hemispheres and between narcolepsy and controls. Means, standard deviations, and estimates from the regression model are reported in Table 3.

We did not detect a significant difference in the DTI‐ALPS index between the left and right hemisphere (*p* = 0.683; Figure S2) or AP and PA phase‐encoding directions (*p* = 0.430). Consequently, for each participant, an average of the left and right hemisphere (*bilateral average*) was calculated for subsequent analyses. From the comparison of the bilateral average DTI‐ALPS index, no significant differences were found between people with narcolepsy type 1 and matched controls (*p* = 0.461; Figure 1, Table 3). The DTI‐ALPS index did also not significantly correlate with ESS (*p* = 0.154; Figure 3), PSG total sleep time (*p* = 0.551), sleep efficiency (*p* = 0.191), or percentage of slow wave sleep (*p* = 0.775; Figure S3a–c).

**TABLE 3:** DTI‐ALPS index in narcolepsy and controls (*n* = 23).

	DTI‐ALPS index		Regression model			
	Narcolepsy (mean ± SD)	Controls (mean ± SD)	β estimate	*t* value	Cohen's *D*	*p* value
Left hemisphere	1.44 ± 0.15	1.43 ± 0.17	0.01	0.11	0.91	0.913
Right hemisphere	1.46 ± 0.12	1.38 ± 0.16	0.08	1.28	0.53	0.214
Bilateral average	1.45 ± 0.12	1.41 ± 0.14	0.04	0.75	0.31	0.461

*Note*: Regression models predicted DTI‐ALPS index (see first column) based on group (narcolepsy/controls) and did not include any covariates. β estimate reflects unstandardised β; *p* value is uncorrected for multiple testing; Cohen's D is tested controls, narcolepsy.

### 3.3 dBOLD‐CSF coupling

**FIGURE 2:**
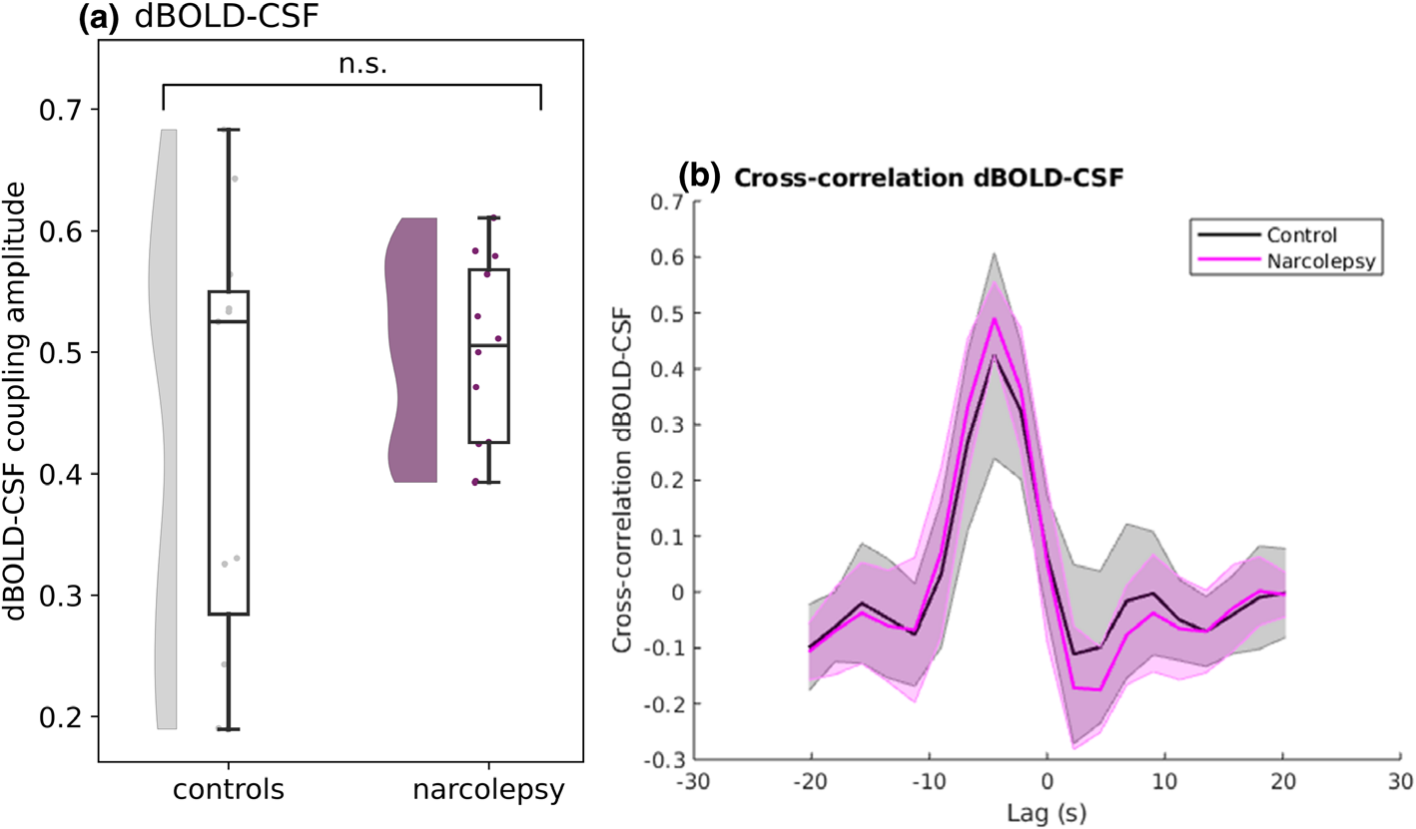
BOLD‐CSF coupling amplitude (a) and coupling curve (b). (a) Quantification of retrieved amplitudes for narcolepsy (purple) and controls (grey). (b) Mean ± SD dBOLD‐CSF coupling curve for narcolepsy (pink) and controls (grey). The peak closest to zero has an average amplitude and lag of 0.50 and −4.13 s (narcolepsy) and 0.43 and −4.09 s (controls). Means, standard deviations, and estimates from the regression model are reported in Table 4.

**FIGURE 3:**
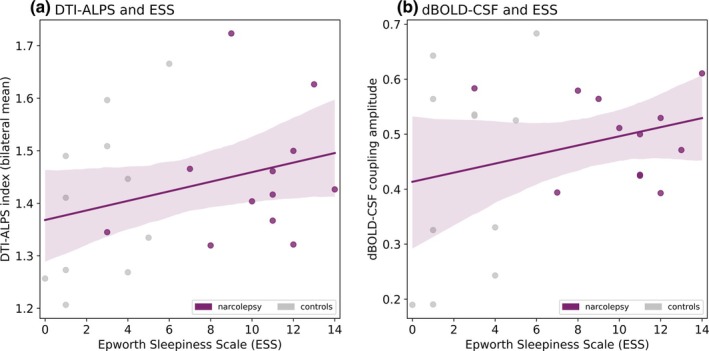
DTI‐ALPS (a) and dBOLD‐CSF coupling (b) correlated with Epworth Sleepiness Scale, a measure of excessive daytime sleepiness. Regression lines are drawn based on data points from narcolepsy and controls combined. DTI‐ALPS: Pearson's *r* = 0.31, *p* value = 0.154; BOLD‐CSF: Pearson's *r* = 0.27, *p* value = 0.208. ESS, Epworth Sleepiness Scale.

**FIGURE 4:**
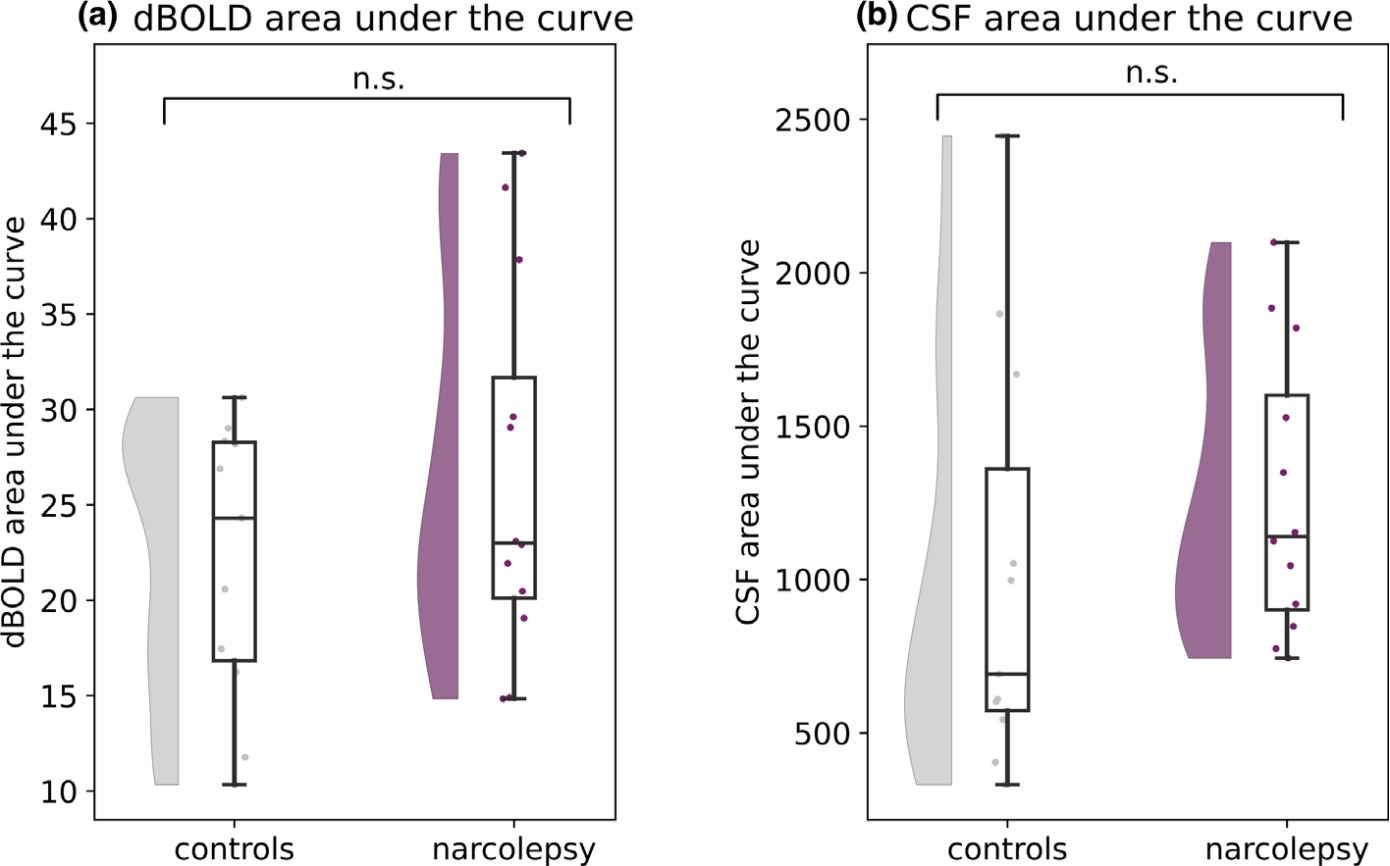
Signal fluctuations in the dBOLD (a) and CSF (b) signal. Area under the curve (AUC) is shown for normalised, detrended, and filtered dBOLD and CSF time series. The AUC did not significantly differ between people with narcolepsy and controls for the dBOLD (*p* = 0.239), as well as the CSF time series (*p* = 0.304).

We explored possible effects of CSF ROI anatomical location on the coupling strength, by rating and dividing the participant images into three categories: fourth ventricle (*n* = 6), lower cerebellum (*n* = 11), and central canal (*n* = 6). The dBOLD‐CSF coupling amplitudes did not differ significantly between the three locations and were pooled for the group comparison (fourth ventricle‐lower cerebellum, *p* = 0.908; fourth ventricle‐central canal, *p* = 0.382; lower cerebellum‐central canal, *p* = 0.323; Figure S4). Between people with narcolepsy type 1 and matched controls, no significant difference was found for dBOLD‐CSF coupling amplitude (*p* = 0.263; Figure 2, Figure S5, Table 4). We also did not observe a significant correlation between the dBOLD‐CSF coupling amplitude and the ESS (*p* = 0.208; Figure 3), PSG total sleep time (*p* = 0.679; Figure S3d), or percentage of slow wave sleep (*p* = 0.889; Figure 3f). A significant correlation was identified between dBOLD‐CSF coupling amplitude and PSG sleep efficiency (*r* = 0.63, *p* = 0.029; Figure S3e). When investigating the dBOLD and CSF signals separately, no differences were found in the area under the curve between people with narcolepsy and controls (dBOLD, *p* = 0.239; and CSF, *p* = 0.304; Figure 4, Table 4).

**TABLE 4:** dBOLD‐CSF coupling in narcolepsy and controls (*n* = 23).

	Regression model					
	Narcolepsy (mean ± SD)	Controls (mean ± SD)	β estimate	*t* value	Cohen's *D*	*p* value
dBOLD‐CSF coupling						
Amplitude	0.50 ± 0.07	0.43 ± 0.17	0.07	1.15	0.48	0.263
Lag (s)	−4.13 ± 0.84	−4.09 ± 1.61	NA	NA	NA	NA
Area under the curve						
dBOLD	26.56 ± 9.44	22.16 ± 6.93	4.41	1.21	0.51	0.239
CSF	1274.05 ± 442.28	1019.16 ± 653.75	254.90	1.05	0.44	0.304

*Note*: Regression models predicted dBOLD‐CSF coupling amplitude based on group status (controls as reference) and did not include any covariates. β estimate reflects unstandardised β; *p* value is uncorrected for multiple testing; Cohen's D is tested (narcolepsy, controls).

### 3.4 DTI‐ALPS and dBOLD‐CSF coupling

**FIGURE 5:**
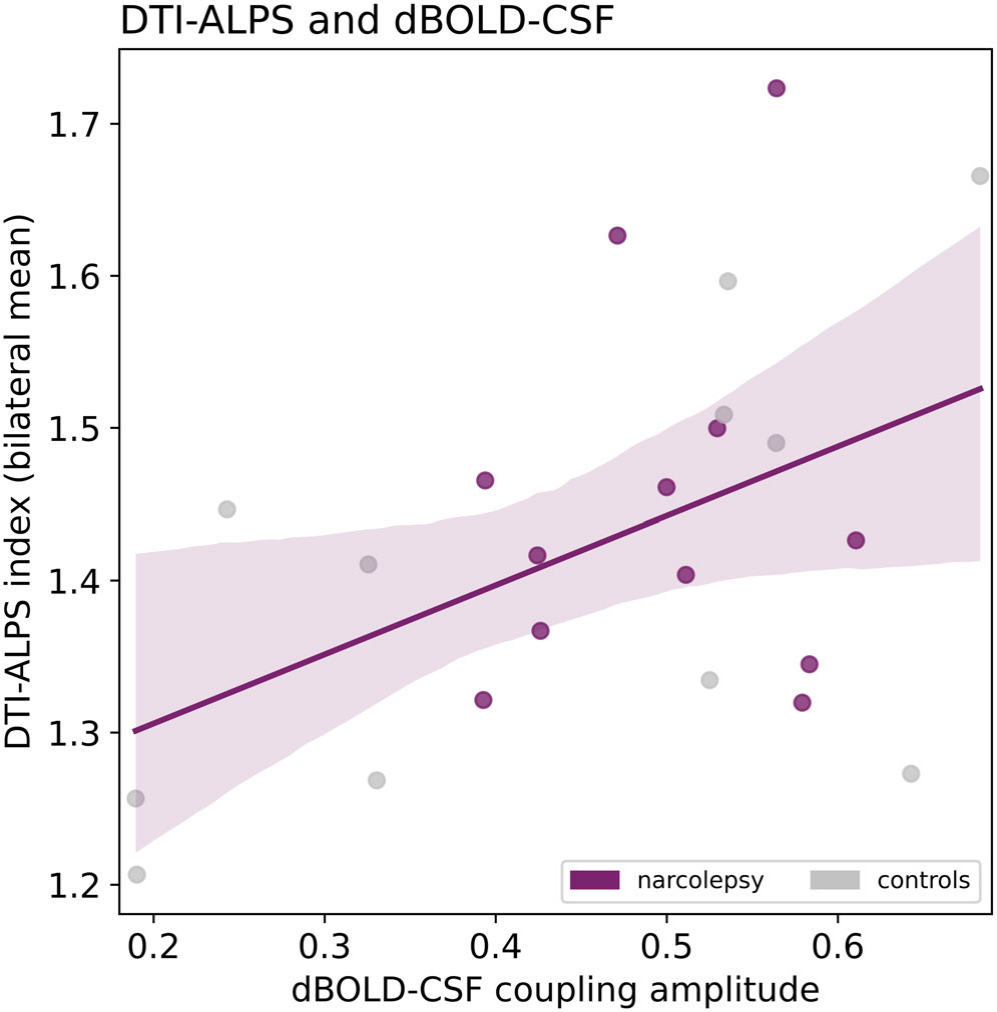
Correlation between DTI‐ALPS index and dBOLD‐CSF coupling amplitude. Higher DTI‐ALPS index (y) is significantly correlated to larger dBOLD‐CSF amplitude (x). Pearson's *r* = 0.46, *p* value = 0.025.

The Pearson's correlation between the two surrogates of brain clearance appeared significant with a positive correlation coefficient (i.e. higher DTI‐ALPS index relates to a higher dBOLD‐CSF coupling amplitude; *p* = 0.025, *r* = 0.46; Figure 5).

## 4 DISCUSSION

In summary, we did not find significant differences between narcolepsy type 1 and healthy controls for either of the surrogate brain clearance measures, nor significant relations with excessive daytime sleepiness. The identified values were similar to previous research for both the DTI‐ALPS index (here: 1.1 to 1.8; Taoka et al., 2017: 0.8 to 1.9) and dBOLD‐CSF coupling amplitude (here: 0.2 to 0.7; Fultz et al., 2019: peak of averages at 0.59) and lag (here: −3.6 s; Fultz et al., 2019: −1.8 s), suggesting reproducibility across different study populations. The correlation between the DTI‐ALPS index and dBOLD‐CSF coupling amplitude was positive and significant (*p* = 0.025), indicating that participants with a high DTI‐ALPS index were also likely to have a high dBOLD‐CSF coupling amplitude, suggesting that both measures might originate from the same underlying physiology. Altogether, this study does not provide evidence for altered brain clearance in narcolepsy type 1 during wake, based on the surrogate MRI measures investigated. However, we want to emphasise that the two surrogate markers have clear limitations in their relation with brain clearance and the absence of significant differences should not be interpreted as an absence of brain clearance alterations in this population. We will discuss these limitations below in more detail.

### 4.1 Altered brain clearance in narcolepsy?

#### 4.1.1 DTI‐ALPS

The absence of group differences in the DTI‐ALPS index aligns with findings from one of the two previous applications of this method in narcolepsy (Gumeler et al., 2023). The authors from this study did not find between‐group differences between narcolepsy type 1 and 2 combined (*n* = 25), compared with controls (*n* = 11). However, Hu et al. (2024), who performed the analysis in a larger dataset, identified a lower DTI‐ALPS index bilaterally in narcolepsy type 1 (*n* = 31) compared with controls (*n* = 23). The DTI‐ALPS index did not significantly correlate with the ESS. In the current study, the correlation between the DTI‐ALPS index and ESS or any of the PSG measures also appeared non‐significant. Whereas the same dataset showed significant group differences on several diffusivity outcome measures in a previous investigation (Gool et al., 2019), it is likely that subtle differences in the DTI‐ALPS index, as well as relations with clinical outcomes, were not detected here due to limited statistical power.

#### 4.1.2 dBOLD‐CSF coupling

We did not find significantly altered dBOLD‐CSF coupling in narcolepsy type 1, contrary to our expectations. As our findings can only be compared with applications in other populations, it remains unclear whether dBOLD‐CSF coupling is truly unaltered in narcolepsy type 1. Previous studies showed a lower coupling strength in neurodegenerative disorders (Han, Chen, et al., 2021; Han, Brown, et al., 2021;Wang et al., 2023), characterised by large brain changes including protein accumulations and impaired vascular reactivity. In the absence of these significant brain alterations, that are likely linked to brain clearance, it is possible that people with narcolepsy type 1 do not show altered dBOLD‐CSF coupling. Yet, as we know from previous work in healthy individuals that dBOLD‐CSF coupling is stronger during sleep (Fultz et al., 2019), we speculate that people with narcolepsy might show normal coupling during wake, but altered coupling during sleep. Notably, we identified a significant positive correlation between dBOLD‐CSF coupling amplitude and PSG sleep efficiency, possibly suggesting that more effective and uninterrupted sleep may facilitate stronger neurovascular‐CSF coupling. In contrast, the PSG total sleep time was not significantly correlated, which may imply that sleep duration is less important than sleep efficiency. Although we expected a correlation with the percentage of slow wave sleep, the lack of correlation perhaps implies that the specific characteristics of slow waves, such as their amplitude or timing, may influence neurovascular‐CSF coupling more than the global percentage of slow wave sleep. Given the substantial time interval between PSG and MRI measurements and the small sample size, these correlations should be viewed as exploratory and interpreted with caution.

#### 4.1.3 Other MRI‐based measures

While the MRI‐based surrogates investigated in this study have seen limited application in narcolepsy, it is worth noting that another measure has been studied that is loosely associated with brain clearance and related physiological processes. The findings from this study may provide some contextual insight, but should be interpreted with caution, as the exact role of this measure in relation to brain clearance is not yet fully understood. Järvelä and colleagues compared brain pulsations measured with ultrafast functional MRI between narcolepsy type 1 (*n* = 23) and controls (*n* = 23; Järvelä et al., 2022). They found a greater variance of pulsations in the low‐frequency band and lower variance in the respiratory band in narcolepsy compared with controls, most prominent in the ascending arousal network. The authors propose that these results imply impaired parenchymal and ventricular CSF flow, likely related to a lack of hypocretin signalling on the downstream ascending arousal network and cortical regions.

#### 4.1.4 Hypocretin, sleep, and amyloid‐β

Both animal and human studies have indicated a relation between hypocretin signalling, sleep, and amyloid‐β in the brain (Berteotti et al., 2021). Strikingly, lower levels of hypocretin, as observed in narcolepsy type 1, seem to have a neuroprotective effect. This idea is based on Alzheimer mice experiments showing that hypocretin infusion increased amyloid‐β levels in the ISF, whereas hypocretin antagonists or hypocretin knockouts showed lower levels and less plaque formation (Kang et al., 2009). As these changes in amyloid‐β levels co‐occurred with improved or disrupted sleep, it is proposed that this effect of hypocretin on amyloid‐β metabolism and clearance is solely mediated through the sleep–wake cycle (Berteotti et al., 2021). In humans, it is known that hypocretin and amyloid‐β in the CSF show circadian fluctuations related to each other (Slats et al., 2012). Additionally, significantly higher levels of hypocretin are observed in CSF of people with Alzheimer's disease pathology, and higher levels correlated with insomnia symptoms (Liguori, Nuccetelli, et al., 2016). In animals, as well as humans, beneficial effects of hypocretin antagonists have been observed on sleep, amyloid‐β‐related neurodegeneration, and cognition (Berteotti et al., 2021). Specifically, to narcolepsy, it is proposed that the low hypocretin signalling reduces the risk of amyloid‐β accumulations and thus Alzheimer's disease (Berteotti et al., 2021). Evidence, so far, is sparse, with one post‐mortem study showing a similar prevalence of amyloid‐β neuropathology as in the general population (Alzheimer's disease was present in 4 out of 12 cases of narcolepsy type 1; Scammell et al., 2012) and one PET study showing a lower brain amyloid burden and lower pathological amyloid accumulation in individuals with narcolepsy (Gabelle et al., 2019). The role of brain clearance in this interplay remains poorly understood, leaving a critical gap in research. Future studies are needed to explore whether hypocretin influences brain clearance processes directly or if its effects are entirely mediated through changes in the sleep–wake cycle.

All in all, it remains to be further explored whether brain clearance is altered in narcolepsy type 1. As the investigation of brain clearance in narcolepsy is only in its infancy, future research should aim to further explore the hypothesis of altered brain clearance in narcolepsy type 1, and consider other central disorders of hypersomnolence, and the importance of comparing different states of consciousness. With more research on brain clearance in narcolepsy type 1, we aim to determine whether brain clearance alterations are present, and if so, to better understand whether they are intrinsic to the disease, a consequence of hypocretin deficiency, or a result of disrupted sleep.

### 4.2 Methodological considerations

#### 4.2.1 Data variability

The range of data points from the two groups in this study shows a distinct pattern. For the dBOLD‐CSF coupling amplitude, people with narcolepsy type 1 show a narrower range compared with the controls (Figure 2). A similar, although less strong, pattern is observed for the CSF area under the curve (Figure 4b). This pattern could originate from the narcolepsy group being very homogenous, with similar pathology and corresponding brain correlates.

#### 4.2.2 DTI‐ALPS

The DTI‐ALPS method (Taoka et al., 2017) is recent, but widely applied in the past years. The main reason for this popularity is likely related to the possibility of applying the method retrospectively, on already processed DTI metrics. In addition, the methodological procedure is easy and not highly time‐consuming. The validity of the DTI‐ALPS index, however, has been debated, with a focus on what the index measures (Ringstad, 2024; Taoka et al., 2024). Without violation of the underlying assumptions, the index supposedly reflects perivascular fluid flow in a small region of brain parenchyma next to the lateral ventricle, in the perivascular channels surrounding the deep medullary veins. The measure is thus highly specific to a small area of the brain and does not present an accurate reflection of CSF‐mediated clearance at the whole‐brain level. Regional brain clearance effects, as suggested in previous studies (Eide et al., 2021), would therefore not be captured in the DTI‐ALPS index. The assumed unique position of the projection and association fibres in relation to the veins, perpendicular in all directions, can also be questioned. In this study, similar to many other applications of the DTI‐ALPS method, the absence of an image visualising veins (i.e. susceptibility‐weighted image), led us to draw the regions of interest without confirming the presence of underlying deep medullary veins. Finally, possible violation of other assumptions, as the sequence is not CSF‐ nor ISF specific, could result in the DTI‐ALPS index not accurately representing perivascular fluid flow, but rather residual diffusion, tissue motion, slow blood flow, or white matter alterations. The doubts surrounding the DTI‐ALPS method led us to perform a multi‐perspective approach in this study, which allowed for a comparison of the DTI‐ALPS outcomes with the dBOLD‐CSF coupling analysis.

#### 4.2.3 dBOLD‐CSF

In contrast to the DTI‐ALPS method, the dBOLD‐CSF analysis explores the forces driving CSF oscillations by measuring the coupling between cortical grey matter fluctuations and CSF flow at the level of the fourth ventricle. Whereas higher coupling has been observed during sleep, earlier studies confirmed that this coupling can also be observed (to a smaller extent) in awake individuals (Han, Chen et al., 2021; Han, Brown, et al., 2021; Wang et al., 2023). The dBOLD‐CSF coupling represents one of the drivers of CSF flow, neurovascular activity, but in relation to CSF only measured at the level of the fourth ventricle (i.e. reflective of large scale CSF mobility which can be different from CSF‐mobility close to the neurons where neuronal waste is produced). The question remains whether this measure, especially when altered in clinical populations, can be translated to provide information about aspects of clearance inside the entire brain, for example perivascular fluid flow.

#### 4.2.4 Complementary surrogates of brain clearance

The significant positive correlation between the DTI‐ALPS index and dBOLD‐CSF coupling suggests that both surrogates capture (parts of) the same or similar processes. Based on the proposed mechanisms behind the measures, we hypothesise that the DTI‐ALPS index and dBOLD‐CSF coupling offer different perspectives of the same underlying physiology, complementing each other methodologically. Future investigations should aim to identify the exact relationships between the two measures.

#### 4.2.5 Study set‐up and future directions

The current study acquired images with a TR of 2250 ms, compared with a TR of 367 ms in the study of Fultz et al. (2019). A faster sampling of the functional MRI signal would offer a better temporal resolution to identify the dBOLD‐CSF coupling lag and possible group differences, which was not possible in this retrospective study. One of the main limitations of the current study is its small sample size. Future work in larger datasets could point out whether the absence of brain clearance alterations in narcolepsy type 1 as reflected by the DTI‐ALPS and dBOLD‐CSF coupling, can be replicated. Possibly, subtle alterations would only be captured by a better‐powered study. Moreover, sleep parameters were not assessed in the control group, which limits the ability to directly compare the relationship between sleep and brain clearance surrogates across groups. Future studies should include detailed sleep assessments in both people with NT1 and controls to enable a more comprehensive understanding of these relationships. If differences in these measures are detected in future studies, an interesting focus would be to investigate and compare different subtypes of hypersomnolence disorders: narcolepsy type 1, type 2, and idiopathic hypersomnia. Additionally, to gain insights into the influence of sleep on the available surrogates, future investigations could acquire MR images at different times of day, during different states of consciousness (wake, REM and NREM stages) and after sleep deprivation. Finally, other MRI‐based surrogates of brain clearance could be explored in narcolepsy. Studies have attempted to estimate CSF and ISF flow or exchange using: phase‐contrast MRI (Kim et al., 2016; Korbecki et al., 2019); variants of arterial spin labelling (ASL; Taoka & Naganawa, 2020; Petitclerc et al., 2021); diffusion‐based imaging (Örzsik et al., 2023; Shao et al., 2019; Wong et al., 2020; Zhang et al., 2012), motion‐prepared imaging (Harrison et al., 2018; Hirschler et al., 2024) or magnetisation transfer ASL (Li et al., 2022). These measures potentially capture a different aspect, which could improve our understanding of whether and how brain clearance is affected or involved in narcolepsy type 1. Beyond these MRI‐based techniques, non‐invasive methods such as functional near‐infrared spectroscopy (fNIRS; Ilvesmäki et al., 2024) and invasive methods such as spinal CSF measurements have been explored as surrogates for brain clearance. Additionally, innovative approaches such as an inner‐ear device that is claimed to measure parenchymal resistance offer a novel perspective (Dagum et al., 2024), although further research and validation are necessary to establish their reliability and interpretability in this context.

## 5 CONCLUSION

To conclude, the hypothesis of altered brain clearance in narcolepsy type 1 is not supported by evidence from the current study. Future work should aim to investigate other (surrogate) measures of brain clearance to better understand the possible role of altered brain clearance in narcolepsy type 1, especially during non‐wake conscious states, and in comparison with other central disorders of hypersomnolence.

## AUTHOR CONTRIBUTIONS

**Eva M. van Heese:** Writing – original draft; investigation; formal analysis; data curation; visualization. **Jari K. Gool:** Conceptualization; writing – review and editing; investigation; data curation. **Gert Jan Lammers:** Supervision; writing – review and editing. **Ysbrand D. van der Werf:** Writing – review and editing; supervision; funding acquisition; conceptualization; methodology. **Matthias J. P. van Osch:** Methodology; writing – review and editing; supervision; software. **Rolf Fronczek:** Writing – review and editing; supervision. **Lydiane Hirschler:** Conceptualization; investigation; writing – review and editing; methodology; validation; visualization; software; supervision.

## FUNDING INFORMATION

This work was supported by Leiden University Fund/Den Dulk‐Moermans Fund.

## CONFLICT OF INTEREST STATEMENT

The authors declare no financial or non‐financial interests.

## Supporting information


**DATA S1** Supporting Information.

## Data Availability

The raw data underlying this article cannot be shared publicly to guarantee the privacy of individuals that participated in the study. Derived data (i.e. outcomes after diffusion/functional MRI processing) will be shared on reasonable request to the corresponding author. The processing pipelines are openly available through github.com/evavanheese/.
